# The Impact of Algorithmic Price Discrimination on Consumers’ Perceived Betrayal

**DOI:** 10.3389/fpsyg.2022.825420

**Published:** 2022-05-17

**Authors:** Zhiyan Wu, Yuan Yang, Jiahui Zhao, Youqing Wu

**Affiliations:** Department of Marketing, School of Management, Shanghai University of International Business and Economics, Shanghai, China

**Keywords:** algorithmic price discrimination, perceived betrayal, perceived price fairness, perceived ease of use, third-degree price discrimination

## Abstract

With the development of artificial intelligence technology, data support is increasing in importance, as are problems such as information disclosure, algorithmic discrimination and the digital divide. Algorithmic price discrimination occurs when online retailers or platforms charge experienced consumers who are purchasing products on their online platforms higher prices than those charged to new consumers for the same products at the same time. The purpose of this paper is to investigate the impact of algorithmic price discrimination on consumers’ perceived betrayal. This paper employed a field experimental method involving two studies. In total, 696 questionnaires were distributed to consumers: 310 for Study 1 and 386 for Study 2. The collected data were analyzed using variance analysis and process analysis methods and SPSS software. Our findings suggest (1) Increased algorithmic price discrimination leads to increased perceived betrayal. (2) Increased algorithmic price discrimination leads to lower perceived price fairness and therefore to increased perceived betrayal among consumers. (3) Higher perceived ease of use of online retailers decreases the impact of algorithmic price discrimination on consumers’ perceived betrayal. We are a small group of researchers focusing on algorithmic price discrimination and integrating algorithmic discrimination into the consumer research field. Our research introduces the concept of consumer perceived betrayal to the field of artificial intelligence. We adopt a field experimental study to examine the impact of algorithmic price discrimination on consumers’ perceived betrayal by introducing variables of perceived price fairness and perceived ease of use.

## Introduction

Online shops can offer different consumers different prices. Such a pricing strategy can lead to advanced forms of price discrimination based on the individual characteristics of consumers, which can be obtained through the use of algorithms to examine consumers’ personal data. In 2000, Amazon differentially priced DVDs based on users’ demographics, shopping histories and online behaviors (e.g., BBC News, 2000). Many consumers were angry about this and raised the issue of fairness (e.g., Krugman, 2000). Amazon hastily made a press release, stating that the company was only conducting an experiment with random discounts, and offered refunds to people who paid prices above the average (Amazon, 2000). McAfee, an antivirus software developer, offered 79.99 dollars to its previous customers who renew their subscriptions but 69.9 dollars to its new customers for the same software in 2013 (Caillaud and Nijs, 2013). Auchan Direct, a French multinational retail group, provides a free delivery to its new consumers but charges extra delivery fees to its existed customers (Caillaud and Nijs, 2013).

Algorithmic price discrimination occurs when online retailers use big data and algorithms to charge repeat (loyal) customers higher prices than those charged to new consumers for the same goods and services during the same time. Such price discrimination is a type of price discrimination through which firms supply a product to different classes of consumers with different characteristics at various prices (Besbes and Lobel, 2015; Siegert and Ulbricht, 2020). For example, in 2008, 51.3% of Chinese consumers encountered this kind of algorithmic price discrimination (Du et al., 2018), this figure suggests that algorithmic price discrimination is becoming prevalent in today’s society. In China facilitated by social media and word of mouth, consumers can quickly find out and discuss this kind of price discrimination once the practices is reported publicly. Many consumers were angry about this and raised the issue of fairness. However, this issue has received limited attention from the academic literature.

Further, Algorithmic price discrimination aims to maximize the profit by pricing the same product differently for different consumers based on their willingness to pay. Therefore, the price of the product varies with consumers’ willingness to pay. The higher the willingness to pay (loyalty) the consumer has, the higher the price they will be charged. In this sense, algorithmic price discrimination is different from the discount or promotion.

If a repeat (loyal) consumer pays a higher price than a new consumer when they purchase the same product at the same time, the repeat consumer feels that the seller is unjust and experiences a sense of betrayal (Gregoire and Fisher, 2008). Acts of betrayal include situations in which customers (buyers) believe that firms (sellers) have lied to them, taken advantage of them, tried to exploit them, violated their trust, cheated, broken their promises, or disclosed confidential information, and they result in disastrous consequences both for the vulnerable party and for the performance of the business relationship as a whole (Kowalski et al., 2003; Leonidou et al., 2018). For example, Lee et al. (2013) suggest that dish failure causes greater betrayal than service failure in the catering industry.

Furthermore, Haws and Bearden (2006) suggest that dynamic pricing led to price unfairness, which would directly perceive betrayal. Lee et al. (2013) indicate that perceived price fairness plays a moderating role in the relationship between dish service failure and perceived betrayal. Baghi and Gabrielli (2021) suggest that a closer relationship leads to a stronger feeling of betrayal, which results in a worsened brand crisis.

Keni (2018) suggests that trust plays a mediating role between perceived ease of use and repurchase intentions regarding online shopping. Ramayah and Ignatius (2005) indicate that perceived ease of use is positively related to the intention to shop online, whereas perceived usefulness is not significantly related to the intention to shop online. Furthermore, they suggest that perceived ease of use is a significant predictor of perceived usefulness. Hansen et al. (2018) suggest that perceived ease of use (from TAM theory) significantly amplifies (positively moderates) the effect of perceived behavioral control (from TPB theory) on the intention to use social networks for transactions. Algorithmic price discrimination is mainly adopted by the big Internet players (apps). Drawing upon the Technology Acceptance Model (TAM, Davis, 1989), we intended to study how the ease of use may help alleviate the negative impacts of price discrimination. According to TAM, ease of use is an important antecedent of consumers’ perception of a technology (the app in our study). When the app is easy to use, consumers are more likely to perceive high quality and better service of the app and less likely to feel betrayal because of the price discrimination. Academically, the buyer-seller relationship involves trust and betrayal.

This research develops and tests a model of how algorithmic price discrimination influences perceived betrayal, incorporating perceived price fairness as a means to understand how consumers feel betrayal, and examines perceived ease of use as a moderator of the relationship between algorithmic price discrimination and betrayal.

This paper has four contributions. Our first contribution is that previous research mainly focuses on positive effects (e.g., convenience and accuracy) of algorithm, but this paper explores the negative effect of algorithm and examine how algorithmic price discrimination negatively influences consumer perception. This is a way to theoretically contribute to price discrimination theory and perceived betrayal theory. Second, this kind of price discrimination is possible because of the big data and algorithms. Compared to Haws and Bearden (2006) who focused on differential prices for the same product in different purchase situations, we manipulated the prices faced by consumers for the same product from the same seller with the same pricing mechanism at the same time. Subjects compared their prices with the price of a new consumer. By doing so, we can study consumer’s perception about price discrimination purely coming from consumers’ willingness to pay calculated from the algorithms. In this way, we also contribute to that this paper shows that perceived price fairness is a key motivational force that leads consumers to feel betrayal in cases of algorithmic price discrimination. Thus, it provides new insights into why and how consumers feel betrayal under such circumstances. We also highlight perceived betrayal as a particularly influential variable among other variables identified by extant research (e.g., anger, dissatisfaction). This also provides theoretical contribution to psychological processes of how the consumers who experienced algorithmic price discrimination feels betrayal.

A third contribution of this research is that it suggests why and how perceived ease of use reduces the negative effect of algorithmic price discrimination on perceived betrayal, which provides ways for online platforms to alleviate the negative effect of algorithmic price discrimination. In this sense, this research explores the perceived ease of use into economic and consumer behavior areas. Finally, our fourth contribution is that we introduce the concept of consumer perceived betrayal to the field of artificial intelligence with our field experiment method, which might provide insights to governments or regulators who would regulate online platforms and reduces the complaints of consumers suffered algorithmic price discrimination. It makes theoretical contributions to algorithmic theory.

## Theoretical Background and Hypothesis

**FIGURE 1:**
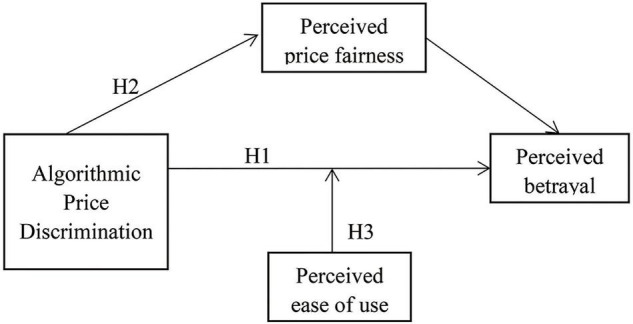
The theoretical framework of this research.

Our model of consumers’ sense of betrayal toward algorithmic price discrimination (see Figure 1) asserts that consumers who experience algorithmic price discrimination feel that fairness norms (perceived price fairness) have been violated; thus, they feel a sense of betrayal (Koehler and Gershoff, 2003). Perceived ease of use has a moderating effect on the relationship between this feeling of betrayal and algorithmic price discrimination. As Figure 1 shows, we test the robustness of our theoretical model by controlling for a variety of factors, including anger and dissatisfaction (Bougie et al., 2003).

### Algorithmic Price Discrimination

Research on algorithmic price discrimination has drawn attention from the areas of law, economics and management. Algorithmic price discrimination is firmly grounded in price discrimination. Price discrimination stems from the fact that firms often price products in various ways to increase revenue (Bitran and Caldentey, 2003; Espinet et al., 2020). Price discrimination is applied across various industries, including the airline (Berry and Jia, 2010; Li et al., 2014; Czerny and Zhang, 2015), consumer packaged goods (Pancras and Sudhir, 2007), perishable storable product (Herbon, 2016), storable goods (Hendel and Nevo, 2013), durable goods (Chevalier and Kashyap, 2019), leisure ticket (Leslie, 2004), and hospital (Grennan, 2013) industries.

Extant literature shows that price discrimination occurs at three levels. First-degree discrimination, which is focused on customers’ willingness to pay, is employed by many firms in the retailing sector to maximize revenue (e.g., Hinz et al., 2011; Jiang et al., 2020; Liu et al., 2020). For example, Hinz et al. (2011) reveal that the use of a name-your-own-price (NYOP) mechanism can increase profits and transactions and that an adaptive threshold price can increase profits by over 20% without decreasing customer satisfaction.

Second-degree price discrimination is an effective way to increase revenue where customers can self-identify or self-select to buy at certain prices; for example, a customer may obtain special benefits and increase his or her status for a higher price (e.g., a first-class airline seat for a higher price or a theater ticket with popcorn for a higher price; Leslie, 2004; Khan and Jain, 2005). Khan and Jain (2005) examined the impact of two price discrimination mechanisms on retailer profitability: quantity discounts based on package size (second-degree price discrimination) and store-level pricing or micromarketing (third-degree price discrimination). They found that a combination of second- and third-degree price discrimination generates the greatest profits, and the inclusion of second-degree price discrimination contributes more to retailer profitability than that of third-degree price discrimination.

Third-degree price discrimination involves purchases made by different classes of consumers with different characteristics within a single product category at various price points. For example, the online travel agency HotelTonight and Orbitz.com offer tailored prices to their users according to their locations and based on whether they are Mac or PC users (Howe, 2017). If existing regulations are in place, third-degree price discrimination can benefit both customers and firms (Armstrong and Vickers, 2001). For instance, a customer who believes that a product is high quality and can afford a higher price is willing to pay more for it than one who believes that the product is low quality. In this way, firms can obtain more revenue and profit. For example, restaurant customers are willing to pay premium prices for special tables with better views or higher levels of service (Borgesius and Poort, 2017; Lin, 2020).

However, price discrimination has some problems, particularly in relation to unfair pricing. For instance, a wide range of online platforms hold increasingly detailed information about their users, including demographic data (e.g., age, location, gender) and behavioral data (e.g., browser history, device type, purchase prices, and times), which can result in price discrimination in the case of monopolies (Esteves and Cerqueira, 2017; Townley et al., 2017; Esteves and Resende, 2019). This is algorithmic price discrimination. Hindermann (2018) notes that algorithmic price discrimination can be based on user characteristics, technical characteristics, and/or location characteristics.

In this paper, we examine how repeat consumers who are charged prices that are higher than those charged to new users for the same product at the same time. At first stage, these repeater consumers do not know that they are charged higher prices than that of the new users, but at the second stage they found that they are charged higher price for the same products at same time, which they are not willing to pay for from the new users. With the availability of consumer information, first-degree price discrimination is also possible. Algorithmic price discrimination is related to both first-degree price discrimination and third-degree price discrimination depends on the level of granularity. In this sense, this paper is to examine a type of price discrimination where how repeat consumers who are charged prices that are higher than those charged to new users for the same product at the same time experience a sense of betrayal by introducing the concept of perceived price fairness.

### Perceived Betrayal

This research argues that the concept of perceived betrayal illustrates the impact of algorithmic price discrimination on repeat customers. Perceived betrayal refers to the fact that a consumer believes that “a firm has intentionally violated what is normative in the context of their relationship” (Gregoire and Fisher, 2008; p 250).

Much of the previous research on betrayal examines the contexts of close relationships (Finkel et al., 2002), employee-employer relationships (Elangovan and Shapiro, 1998), and business relationships (Hannon et al., 2010). The findings of these studies show that acts of betrayal break the “rules of the game” that govern relationships and violate norms of honesty, decency and fairness. In business contexts, acts of betrayal include situations in which customers (buyers) believe that firms (sellers) have lied to them, taken advantage of them, tried to exploit them, violated their trust, cheated, broken promises, or disclosed confidential information, and they result in disastrous consequences both for the vulnerable party and for the performance of the business relationship as a whole (Kowalski et al., 2003; Leonidou et al., 2018).

As its definition shows, betrayal occurs within the context of a relationship, and this makes this construct relevant in typical situations. The relational foundation of betrayal serves as an essential distinction from other existing variables, such as dissatisfaction and anger (Smith et al., 1999; Bougie et al., 2003). Betrayal is associated with the norms regulating a relationship, whereas anger and dissatisfaction may not reference any relational context.

Based on the conceptual differences shown above, our model suggests that betrayal acts as a major motivating force that drives consumers to restore fairness. Specifically, our model supposes that intense algorithmic price discrimination makes consumers perceive violations of their normative expectations, that is, experience perceived betrayal; the higher the degree of algorithmic price discrimination is, the stronger the degree to which the involved consumer believes that the enterprise is violating justice (Bougie et al., 2003; Gregoire and Fisher, 2008); Formally, we suggest the following:

**H1:** The intensity of algorithmic price discrimination positively affects consumers’ perceived betrayal.

### Perceived Price Fairness

Algorithmic price discrimination tends to make repeat consumers perceive unfairness, resulting in a sense of betrayal. Fairness refers to the extent to which outcomes are deemed reasonable and just. Prices that compare favorably with the reference point are deemed fair (Xia et al., 2004). A reference price can be a price paid by someone else to buy the same product, a price offered by another merchant for the product, or a price paid by the focal consumer in the past for the product (Campbell, 1999). This study focuses on interpersonal price differences because they are the most common reference prices used by consumers to evaluate price fairness (Kwak et al., 2015). Haws and Bearden (2006) find that when consumers find that they are paying more than other consumers, they experience a higher sense of price unfairness and lower perceived price fairness than they do when they encounter price differences across various stores and different times. This perceived price unfairness leads to increased perceived betrayal and public complaints and boycotts. Therefore, algorithmic price discrimination reflects firms’ violations of the norms applicable to interactions with consumers, and it is an embodiment of their price fairness violation. When repeat consumers find that they are paying more than new customers when they buy the same product at the same time on an e-commerce platform, they feel that they are facing algorithmic price discrimination and therefore experience a higher sense of price unfairness, which results in decreased perceived price fairness; this in turn increases their sense of perceived betrayal. Formally, we suggest the following:

**H2:** Increased algorithmic price discrimination leads consumers to experience decreased price fairness, which in turn leads to higher perceived betrayal.

### Perceived Ease of Use

Venkatesh (2000) indicates that external and internal control (e.g., buyer-seller relationships), intrinsic motivation (playfulness), and emotions are anchors that determine early perceptions about the ease of use of a new system. Buyer-seller relationships involve trust and betrayal. Keni (2018) suggests that trust plays a mediating role between perceived ease of use and repurchase intentions in the context of online shopping. Ramayah and Ignatius (2005) indicate that perceived ease of use is positively related to the intention to shop online, whereas perceived usefulness is not significantly related to the intention to shop online. They further suggest that perceived ease of use is a significant predictor of perceived usefulness. Hansen et al. (2018) suggest that perceived ease of use (from TAM theory) significantly amplifies (positively moderates) the effect of perceived behavioral control (from TPB theory) on the intention to use social networks for transactions.

Davis (1989) defined perceived ease of use as the degree to which the information format is unambiguous, clear or readable. Perceived ease of use comprises all users experience elements relating to the ease with which users can learn, feel clearly, understandably, easily, flexibly to use. Jahangir and Begum (2008) observe that perceived ease of use exerts a significant impact on the process of user acceptance in relation to electronic banking systems. Due to the large number of e-commerce platforms with very mature technologies, this study views perceived ease of use as a main factor influencing consumers’ trust and thus influencing consumers’ platform choices. Perceived ease of use refers to whether it is easy for users to use the functions of a service platform.

Algorithmic price discrimination makes consumers perceive violations of their normative expectations and thus experience perceived betrayal. The higher the degree of algorithmic price discrimination is, the stronger the degree to which the involved consumer believes that the enterprise is violating justice (Gregoire and Fisher, 2008) and therefore produce the feeling of betrayal. Perceived ease of use focuses on user experiences. Better customers experience generally leads to higher customer *satisfaction* (Sharma and Chaubey, 2014) and trust (Garbarino and Johnson, 1999; Hansen et al., 2018). When consumers experience better ease-of-use, they are satisfied with the platform and perceive it as trustworthy and friendly. Accordingly, their feeling of justice violation reduces and so is the impact of algorithmic price discrimination on the feeling of betrayal. When the platform is not easy to use, frustrated consumers feel stronger justice violation from price discrimination and therefore strengthen the negative impacts of price discrimination on perceived betrayal. Formally, we suggest the following:

**H3**: An increase in consumers’ perceived ease of use of an e-commerce service platform reduces the impact of algorithmic price discrimination on their perceived betrayal.

## Study 1

This experiment was designed mainly to test the main effect of algorithmic price discrimination on consumers’ perceived betrayal (H1) and the mediating role of perceived price fairness in this relationship (H2). Pretest 1 is to show why we use the Meituan platform as our research materials. We also followed the price discrimination description of Hindermann (2018) to develop our measure of algorithmic price discrimination through pretest 2. We further followed Gregoire and Fisher’s (2008) five-item perceived betrayal scales and Haws’ (2006) three-item perceived price fairness scale.

### Pretest 1

The main purpose of Pretest 1 was to identify the apps on which consumers often encounter the use of big data to charge repeat customers higher prices and algorithmic price discrimination. A total of 30 informants took part in the test, and their core task was to write down the names of the web platforms on which they had been charged higher prices as repeat consumers (algorithmic price discrimination). The results of the data analysis showed that the 8 most common apps were Meituan, Didi, Ctrip, Taobao, JD.com, Fliggy, Qunar, and Pinduoduo, and the Meituan platform ranked first (11/30). Thus, our two experiments used the Meituan platform as an experimental setting. Study 1 used the hotel reservation service of the Meituan platform as the target platform for its experiment. The services offered on the platform included takeaways, hotels, shows/movies, tours, airline tickets, taxis, and fitness/sports activities, of which hotel reservations (11/30) and takeaways (9/30) exhibited the highest probability of involving higher charges for repeat consumers. In addition, the most frequently given descriptions were “As a repeat consumer, I pay more for the same product at the same time than new customers on the Meituan app; This price difference is due to the Meituan platform’s use of using big data to charge repeat consumers more than new consumers, which seriously affects my interests; I think that’s how the Meituan platform uses algorithms to discriminate against existing customers.”

### Pretest 2

Pretest 2 was used to test for the existence of algorithmic price discrimination and measure its intensity. Based on the price discrimination scale of Hindermann (2018), the informants of this test are asked to evaluate the price differences induced by the Meituan algorithm, i.e., you believe that on the Meituan platform: (1) There is a price difference, with repeat consumers paying more than new customers (*M* = 5.60); (2) The price difference between repeat consumers and new customers is caused by the Meituan platform’s use of artificial intelligence algorithms to charge repeat customers more than new consumers (*M* = 5.54); (3) This price difference seriously affects your personal rights and interests (*M* = 5.69); (4) This is price discrimination against repeat consumers (*M* = 5.60). A 7-point Likert scale was used, with 1 meaning “totally disagree” and 7 meaning “totally agree.” The reliability coefficient of algorithmic price discrimination was 0.844, and the KMO value of our validity analysis was 0.811, which shows that both the reliability and validity of this test were good.

### Method

The study questionnaire was deployed on the morning of December 15, 2020. We recruited students who had recently planned to travel using the Meituan platform, and 310 of these informants were randomly selected for a centralized experiment. They were given questionnaires and paid RMB10 yuan per person to complete them (40.61% male, with an average age of 20.73 years). Field experiments were used to control pricing entities. Porat (2020) believes that it is easier for some consumers to accept algorithmic price discrimination if they feel that a personalized pricing factor is involved. In addition, consumer sentiment affects its impact. For example, when a consumer in a bad mood encounters algorithmic price discrimination, he or she may feel angrier and think he or she has been seriously betrayed. In summary, the study measured both customization and consumer emotional states during the experimental process to eliminate the confusing impacts of these two factors.

#### Algorithmic Price Discrimination Measurement

Since our second pretest proved that our manipulation of algorithmic price discrimination was successful, we followed the same procedure to stimulate the price differences. We then measure participants’ feelings of betrayal when they were charged different prices.

#### Perceived Betrayal Measurement

Based on Gregoire and Fisher’s (2008) five-item perceived betrayal scales (X lies to you; X cheats you; X betrays you; X attempts to use your data information; and X abuses your data information), we instructed each participant to indicate his or her degree of perceived betrayal.

#### Perceived Price Fairness Measurement

Based on Haws’ (2006) three-item perceived price fairness scale (I think the price I have paid is fair, reasonable, and just), we instructed each participant to indicate his or her degree of perceived price fairness.

### Procedure

Consumers always find out how much other consumers (e.g., friends, relatives, and family numbers) need to pay toward same product to same online retailers at same time, and discuss the differences with other consumers as this kind of price discrimination often reported in online news. Accordingly, first, the informants opened the Meituan app to determine the number of orders they had made on the app and the amount they had spent at Meituan hotels. Next, the researcher (the first author) registered for Meituan accounts on site with new mobile phone numbers, and the researcher and informants logged into the randomly selected “Presidential Hotel Beijing” (a three-star hotel) simultaneously to search for the price of a superior king-size bedroom from January 21 to 25, 2020. The researcher announced that the price she would have to pay as a new user was RMB 1,863 yuan. The informants wrote down how many times he/she used the Meituan app and reported their own prices, and finally determined the difference between the price they paid and the price paid by the new user.

Next, the informants were asked to answer the 4 questions from Pretest 2 (1 = “totally disagree” to 7 = “totally agree”; algorithmic price discrimination *a* = 0.796).

After that, the informants were asked to answer a series of questions about how they personally felt after being subject to algorithmic price discrimination with reference to the perceived betrayal scale of Gregoire and Fisher (2008) (1 = “totally disagree” to 7 = “totally agree”; perceived betrayal *a* = 0.926).

Then, the informants were asked to answer a series of questions about what they thought regarding the prices they paid based on Haws’ (2006) three-item perceived price fairness scale (1 = “totally disagree” to 7 = “totally agree”; perceived price fairness *a* = 0.790).

Finally, other possible explanatory variables were measured. First, Bar-Gill (2020) suggests that if a firm informs a customer that his or her price is higher than others as a result of personalization and that the customer has a choice, the price difference is the result of personalized pricing. The difference is that personalized pricing is price the personalized product or service for a consumer, while price discrimination is price differently for the same product or service. Therefore, we included item 5: “You think that this kind of price difference where a repeat consumer pays more than a new customer is the result of personalized pricing on the Meituan platform where if a firm informs a customer that his or her price is higher than others as a result of personalization and that the customer has a choice, the price difference is the result of personalized pricing.” We used 7-point scales (1 = “totally disagree” to 7 = “totally agree”; personalized pricing *a* = 0.882).

Then, six items were adopted from the study of Lee and Sternthal (1999) to measure the emotional states of the informants when completing the questionnaire: “cheerful, happy, excited, depressed, disappointed, or angry.” We used 7-point scales (1 = “totally disagree” to 7 = “totally agree”; the emotional state *a* = 0.809). In addition, the demographic characteristics of the informants, such as gender, age, income, etc., were measured.

### Results

A total of 310 valid questionnaires were received in this study. After calculation, the average price difference between the prices paid by the informants and the price provided by the researcher who was a new user (1,863 yuan) was shown to be 226.52. The questionnaires were divided into groups, and those with above-average prices were classified as the high price difference group (170 questionnaires), while those with below-average prices were classified as the low-price difference group (140 questionnaires). The average price is calculated as the mean of the prices wrote down by each participant for the same product at the same time. The results of a one-way variance analysis show that the price discrimination score of the group with high-intensity algorithmic price discrimination (*M*_high_ = 5.71) is significantly higher than that of the low-intensity group [*M*_low_ = 3.73, *F*(1,308) = 143.13, *p* < 0.001], indicating the control success of the algorithmic price discrimination in this study.

**FIGURE 2:**
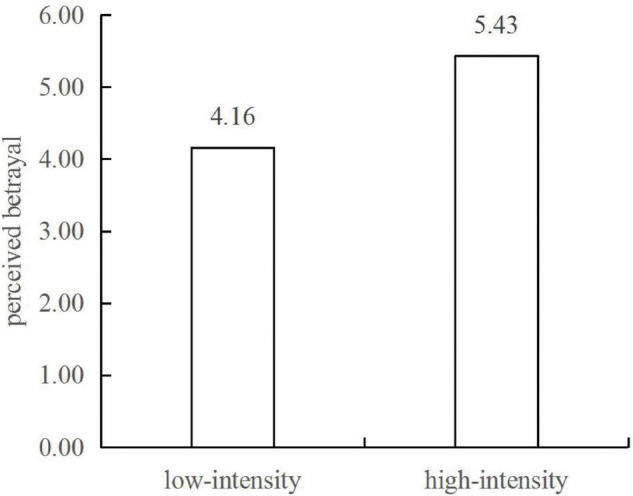
The impact of algorithmic price discrimination on perceived betrayal.

The main effect is that of the intensity of algorithmic price discrimination on perceived betrayal. The results of a one-way variance analysis show that both groups perceive betrayal, but the perceived betrayal of the low-intensity algorithmic price discrimination group (*M*_low_ = 4.16) is significantly lower than that of the high-intensity group [*M*_high_ = 5.43, *F*(1,308) = 30.75, *p* < 0.001; see Figure 2]. This shows that high-intensity algorithmic price discrimination has a greater impact on consumers’ perceived betrayal than low-intensity algorithmic price discrimination. Therefore, H1 is validated.

**FIGURE 3:**
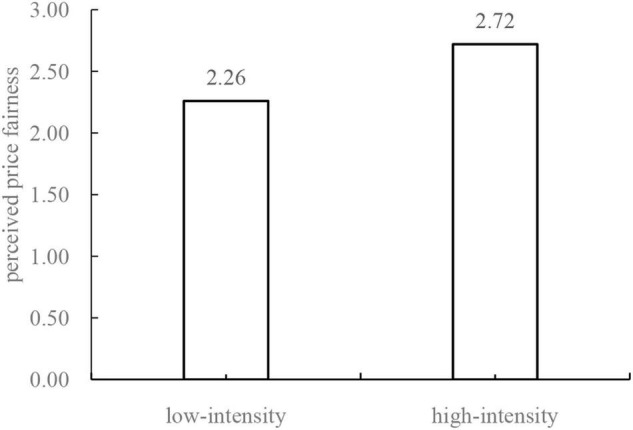
The mediating role of perceived price fairness.

Moreover, we verify the effect of algorithmic price discrimination on perceived price fairness and other possible explanatory variables. The results of a variance analysis show that the perceived price fairness of the low-intensity price discrimination group (*M*_low_ = 2.26) is significantly lower than that of the high-intensity price discrimination group [*M*_high_ = 2.72, *F*(1,308) = 10.45, *p* < 0.01; see Figure 3]. This shows that consumers facing highly intense algorithmic discrimination have lower levels of perceived price fairness than those facing little algorithmic price discrimination, which is consistent with the hypothesis of this study.

If a firm informs a customer that his or her price is higher than others as a result of personalization and that the customer has a choice, the price difference is the result of personalized pricing. Personalized pricing influences the high-intensity algorithmic price discrimination group (*M*_high_ = 1.102) and the low-intensity group [*M*_low_ = 2.134, *F*(1,384) = 0.921, *p* = 0.217]. Moreover, emotional state influences the high-intensity algorithmic price discrimination group (*M*_high_ = 4.066) and the low-intensity group [*M*_low_ = 4.012, *F*(1,384) = 0.207, *p* = 0.325]. There is no significant difference between the evaluations of the high-intensity and the low-intensity algorithmic price discrimination groups of personalized pricing and emotional state, indicating that these two variables cannot explain the main effect of high-intensity algorithmic price discrimination on perceived betrayal. Moreover, the means of the personalized pricing variable in the two groups are considerably less than 4, which indicates that personalized pricing is not a mediating variable of the algorithmic price discrimination relationship; moreover, the mean of emotional state is close to 4, indicating that the informants experience steady emotional states. Therefore, the potential impacts of these two possible factors can be ruled out, and these two variables are not included in the subsequent mediation test.

We used a process analysis to test the mediating effect of perceived price fairness, taking perceived betrayal as a dependent variable, algorithmic price discrimination as an independent variable, and perceived price fairness as a mediating variable. The results show that perceived price fairness affects the impact of algorithmic price discrimination on perceived betrayal with a 95% confidence interval of [-0.5561, -0.3962], β = 1.2733, *SE* = 0.2745, *P* < 0.01. The consumers subject to high-intensity algorithmic price discrimination have lower perceived price fairness and higher perceived betrayal than those in the low-intensity group. Therefore, the results support H2.

### Discussion

This study employs two between-group experiments to verify the main effect of the perceived betrayal of consumers subject to high-intensity algorithmic price discrimination and verifies the mediating effect of perceived price fairness while ruling out the possible impacts of two potential influencing factors: personalized pricing and emotional state. This study reveals the mechanism underlying the impact of algorithmic price discrimination on consumers’ perceived betrayal, and a follow-up study will examine the conditions under which algorithmic price discrimination may alleviate perceived betrayal.

## Study 2

Study 2 is to reduce the negative effect of the algorithmic price discrimination on perceived betrayal, which will give insights for online platforms. The main purpose of experiment 2 was to verify the moderating role of consumers’ perceived ease of use (H3).

### Pretest 3

The first goal of this pretest was to test whether the takeaway service of the Meituan platform and the “PluShine Bok” Songjiang University City store were the most popular takeaway options in Songjiang University City. The results of Pretest 1 show that consumers were most likely to be charged higher prices as repeat consumers on the Meituan platform, and the takeaway service of this platform ranked second in terms of vulnerability to this practice. Therefore, we selected the Meituan Takeaway Service as the setting for Study 2. Moreover, 20 respondents were interviewed regarding the Meituan Takeaway stores they frequented and liked and the corresponding products. “PlusShine Bok” Songjiang University City store ranked first (35%), and the “Shine in Package II” (92.7%), “Shine in Package IV” (83.3%), “Shine in Package VI” (81.5%), and “Shine in Package VII” (75.4%) products were the first, second, third and fourth most popular products, respectively.

The second goal of this pretest was to test the effectiveness of the “PlusShine Bok” Songjiang University City Store and its products “Shine in Package II,” “Shine in Package IV,” “Shine in Package VI,” and “Shine in Package VII” as the service products of concern for this research and to measure the existence and intensity of algorithmic price discrimination in this context. A total of 40 informants took part in the test. We used 7-point scales (1 = “totally disagree” to 7 = “totally agree”; “PlusShine Bok” Songjiang University City Store *M* = 5.43). The informants also thought that (1) there was a price difference, with repeat consumers paying more than new customers (*M* = 5.66); (2) this price difference was caused by the Meituan platform’s use of big data and AI algorithms (*M* = 6.03); (3) this price difference seriously affected their personal rights and interests (*M* = 5.45); this was price discrimination against repeat consumers on the Meituan platform (*M* = 5.79); that the greater this price difference was, the greater the price discrimination against the repeat consumers of Meituan Takeaway was (*M* = 5.85). The leading coefficient of the reliability of algorithmic price discrimination was 0.810, and the KMO value was 0.780; these values indicate good reliability and validity.

### Method

The utilized experimental material was deployed on December 20, 2020 using a two-factor 2 (algorithmic price discrimination: high vs. low) × 2 (perceived ease of use: high vs. low) intergroup design to test the interaction hypothesis given that they dummy coded both variables. We made dummy variable into groups because we adopted the grouping experiment to test interactions of variables. In this way, we ran PROCESS model 1 that we need to dichotomize these variables. The questionnaires were randomly issued to 386 students who had recently used Meituan Takeaway services (43.36% of whom were male; the average age was 20.75), and each of the respondents was paid 10 yuan for completing the questionnaire. This study’s approach to algorithmic price discrimination was similar to that of Study 1, and the same realistic scenario approach was used; however, the experimental products were replaced with takeaways (low-priced service products). Based on the results of Pretest 4, we used the “PlusShine Bok” Songjiang University City Store and four of its products, “Shine in Package II,” “Shine in Package IV,” “Shine in Package VI,” and “Shine in Package VII,” which can be ordered together, as experimental materials. Our approach to controlling the consumers’ perceived ease of use drew mainly on the four items used in the study of Viswanath et al. (2003).

#### Perceived Ease of Use Measurement

Based on Viswanath’s et al. (2003) four-item perceived ease of use scales (it is easy to learn how to use X; the use of X is clear and understandable; it is easy to use X; the use of X is flexible), we instructed each participant to indicate his or her degree of perceiveFFGFfd betrayal.

### Procedure

First, the informants opened the Meituan app to determine the number of orders they had made and the amount of money they had spent on takeaways from Meituan. Then, the researchers (the first author) registered for Meituan accounts on-site with new mobile phone numbers. The researcher and the informants selected the “PlusShine Bok” Songjiang University City Store and clicked to buy the four examined products together, namely, “Shine in Package II,” “Shine in Package IV,” “Shine in Package VI,” and “Shine in Package VII” (see Pretest 3). The researcher announced that as a new user, she would pay a total of 78 yuan. Then, each of the informants wrote down how many times he/she used the Meituan app and reported their own prices, and finally determined the difference between the price they paid and the price paid by the new user.

The informants were asked to imagine being in an actual purchase scenario where there was a difference between the prices paid for the abovementioned service and to answer questions about how they felt. The 4 items from Study 1 were used to measure the intensity of algorithmic price discrimination (α = 0.817), while the 5 items from Study 1 were used to measure perceived betrayal (α = 0.870).

In addition, the informants were asked to report demographic information regarding gender, age, income, etc. Based on Viswanath’s et al. (2003) perceived ease of use scale, the informants’ perceived ease of use was measured (1 = “totally disagree” to 7 = “totally agree”; perceived ease of use α = 0.802).

### Results

In this study, 386 valid questionnaires were collected. After calculation, the average difference between the prices of the informants and the price (78 yuan) provided by the researcher (new user) was shown to be 6.78. The questionnaires were divided into groups; those with above-average prices were classified as the high price difference group (183 questionnaires), while those with below-average prices were classified as the low-price difference group (203 questionnaires). A mean perceived ease of use greater than 4 was considered high, and that with a mean less than 4 was considered low. The 386 questionnaires were then categorized into the following groups: 105 exhibited low-intensity price difference–low perceived ease of use, 98 exhibited low-intensity price difference–high perceived ease of use, 87 exhibited high-intensity price difference–high perceived ease of use and 96 exhibited high-intensity price difference–low perceived ease of use.

The intensity of algorithmic price discrimination was tested with a control. The results of a one-way variance analysis show that the high-intensity algorithmic price discrimination experimental group scored significantly higher (*M*_low_ = 4.62) than the low-intensity algorithmic price discrimination group [*M*_high_ = 5.31, *F*(1,384) = 21.567, *p* < 0.001] in terms of perceived algorithmic price discrimination. This result demonstrates the success of this study in controlling the intensity of algorithmic price discrimination.

**FIGURE 4:**
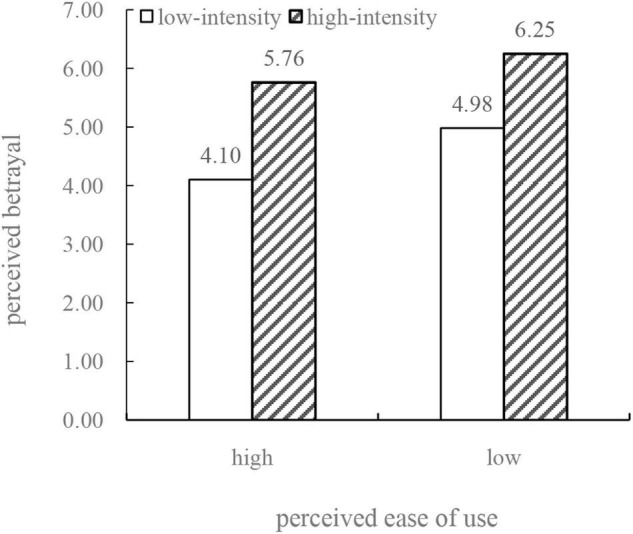
The moderating role of perceived ease of use.

We used the process analysis method (Hayes, 2013, Model 1) to test the moderating effect of consumers’ perceived ease of use. We took perceived betrayal as a dependent variable, algorithmic price discrimination as an independent variable, and perceived ease of use as a moderating variable, and the results show that algorithmic price discrimination has a significant effect on perceived betrayal [*t*(382) = 10.329, *p* < 0.001]. Again, the results support H1. The interaction between algorithmic price discrimination and perceived ease of use has a significant impact on perceived betrayal, *t*(382) = 2.941, *p* = 0.0035 < 0.01. Figure 4 presents the statistical results regarding the mean perceived betrayal scores of the informants with high perceived ease of use and low perceived ease of use in both groups. Figure 4 shows the effect of algorithmic price discrimination on perceived betrayal is moderated by perceived ease of use. Furthermore, this figure shows that among the consumers with high perceived ease of use, the perceived betrayal of high-intensity algorithmic price discrimination (*M*_high_ = 5.76) experienced by the informants with high perceived ease of use is significantly higher than that of the low algorithmic price discrimination group [*M*_low_ = 4.10, *t*(382) = 10.329, *p* < 0.001]. Among the consumers with low perceived ease of use, the difference in the perceived betrayal caused by these two types of algorithmic price discrimination is significantly less than that of the high perceived ease of use group. The mean of the low-intensity algorithmic price discrimination group is 4.98 (*M*_low_ = 4.98), the mean of the high-intensity algorithmic price discrimination group is 6.25 [*M*_high_ = 6.25, *t*(382) = 16.454, *p* < 0.001]. We used a process analysis to test the moderating effect of perceived ease of use, taking perceived betrayal as a dependent variable, algorithmic price discrimination as an independent variable, and perceived ease of use as a moderating variable. The results show that perceived ease of use affects the impact of algorithmic price discrimination on perceived betrayal with a 95% confidence interval of [–0.9558, –0.1899], β = –0.5729, *SE* = 0.2376, *P* < 0.001. The above results show that consumers’ perceived ease of use plays a moderating role in the impact of algorithmic price discrimination on perceived betrayal and that the main effect of high-intensity algorithmic price discrimination is more significant among consumers with low perceived ease of use. Therefore, H3 is validated.

### Discussion

This study’s results support the main effect of high-intensity algorithmic price discrimination on consumers’ perceived betrayal in the product category of takeaways. Venkatesh (2000) indicates that external and internal control (e.g., buyer-seller relationships), intrinsic motivation (playfulness), and emotions are anchors that determine early perceptions about the ease of use of a new system. Buyer-seller relationships involve trust and betrayal. Keni (2018) suggests that trust plays a mediating role between perceived ease of use and repurchase intentions in the context of online shopping. However, we tested the moderating role of perceived ease of use, finding that the main effect of high-intensity algorithmic price discrimination among consumers with high perceived ease of use is significantly reduced. Thus, with an increase in experience, it is expected that system-specific perceived ease of use, while remaining anchored to general beliefs regarding computers and computer use, will adjust to reflect objective usability, perceptions of external control specific to the new system environment, and system-specific perceived enjoyment. Therefore, perceived ease of use is a boundary condition that plays a positive role in high-intensity algorithmic price discrimination.

To sum up, this paper examines how algorithmic price discrimination gives negative impact on consumer’s perceived betrayal, by linking the concept of perceived price fairness and perceived ease of use. This paper uses experimental methods to explore the mechanism underlying the impact of algorithmic price discrimination on consumers’ perceived betrayal by manipulating algorithmic price discrimination. First, Fernandes and Calamote (2016) suggest that unfairness perception is stronger for existing than for new clients, prompting negative attitudinal and behavioral consequences when the former are exposed to disadvantaged conditions in relation to the latter. However, our findings suggest that increased algorithmic price discrimination leads to increased perceived betrayal. It theoretically contributes to price discrimination theory and perceived betrayal theory.

Second, Haws and Bearden (2006) suggested the effects of seller-, consumer-, time-, and auction-based price differences on perceived price fairness and purchase intension. Bolton et al. (2003) indicate that in the context of four-dimensional transaction space of both individual and multiple transactions. Our research finding suggest that compared with consumers who experience less intense algorithmic price discrimination, consumers who experience highly intense algorithmic price discrimination are more likely to exhibit lower levels of perceived price fairness, which in turn produces higher perceived betrayal. It introduces perceived betrayal and perceived price fairness into algorithmic area. It also provides psychological process of when consumers experience algorithmic price discrimination, they will feel price unfairness, and results in feeling of betrayal.

Third, little research brings peeved ease of use into price discrimination and perceived betrayal areas. But this research finding suggests when consumers have higher perceptions of the ease of use of an online service platform, the impact of high-intensity algorithmic price discrimination on their perceived betrayal is lower. Such finding theatrically contribute to perceived ease of use theory.

There are two limitations of the empirical studies. First, this paper employed the lab experimental studies, which could not give the real scenario as field experimental study adopted. Second, the second limitation is that the average age of the informants is 20.75, which showed most of our informants are aged around 20 years old. There are two theoretical limitations of the model. First, this paper did not examine the further behavior after algorithmic price discrimination. second, this paper did not examine how first (algorithmic) price discrimination influences consumer behavior.

## Conclusion

Algorithmic price discrimination causes repeat consumers to perceive betrayal when they find themselves purchasing the same product as new consumers at the same time at higher prices. This study uses experimental methods to explore the mechanism underlying the impact of algorithmic price discrimination on consumers’ perceived betrayal by manipulating algorithmic price discrimination within two different product categories. Our findings are as follows. (1) Increased algorithmic price discrimination leads to increased perceived betrayal; (2) Compared with consumers who experience less intense algorithmic price discrimination, consumers who experience highly intense algorithmic price discrimination are more likely to exhibit lower levels of perceived price fairness, which in turn produces higher perceived betrayal; (3) When consumers have higher perceptions of the ease of use of an online service platform, the impact of high-intensity algorithmic price discrimination on their perceived betrayal is lower.

## Contribution and Implications

This paper explores possible negative effects of algorithm and examine how algorithmic price discrimination negatively influences consumer perception. This is a way to theoretically contribute to price discrimination theory and perceived betrayal theory. Furthermore, this paper shows that perceived price fairness is a key motivational force that leads consumers to feel betrayal in cases of algorithmic price discrimination. Thus, it provides new insights into why and how consumers feel betrayal under such circumstances. We also highlight perceived betrayal as a particularly influential variable among other variables identified by extant research (e.g., anger, dissatisfaction). This also provides theoretical contribution to psychological processes of how the consumers who experienced algorithmic price discrimination feels betrayal.

This paper suggests why and how perceived ease of use reduces the negative effect of algorithmic price discrimination on perceived betrayal, which provides reference for online platforms to reduce the negative effect of algorithmic price discrimination. In this sense, this research explores the perceived ease of use into economic and consumer behavior areas. Additionally, we introduce the concept of consumer perceived betrayal to the field of artificial intelligence with our field experiment method, which might provide insightful reference for governmental regulars who would release regulations to online platforms and therefore reduces the complaints of consumers who suffer this kind of algorithmic price discrimination. It makes theoretical contributions to algorithmic theory.

This paper provides implications for governments to regulate online platforms because high high-intensity algorithmic price discrimination leads to the perceived betrayal of consumers and complains. Further, this study provides a reference for the marketing communication of online platform firms. This study shows that high-intensity algorithmic price discrimination has a strong negative effect on consumers’ perceived betrayal. Therefore, firms should reduce price discrimination as much as possible in their marketing, as this may enhance consumers’ trust in them. In addition, network service platforms can reduce the negative effect of algorithmic price discrimination by making their platforms easier to use because the ease of perceived use is moderated the relationship between algorithmic price discrimination and perceived betrayal.

## Limitation and Future Study

This study has two limitations that can be explored in future studies. First, this article uses the research method of field experiments. Future studies could use secondhand data analyses to test the hypotheses of this study, for example, using crawler software to capture price-related information from user product reviews or conducting content analyses to test the impact of algorithmic price discrimination on consumers’ perceived betrayal. Second, in relation to the development of the dependent variables, this study focuses on the impact of algorithmic price discrimination on perceived betrayal. Moreover, further study should be conducted on follow-up behaviors occurring after the impact of algorithmic price discrimination on perceived betrayal and on how behaviors induced by algorithmic price discrimination can be mitigated while maintaining corporate profits.

## Data Availability Statement

The original contributions presented in the study are included in the article/supplementary material, further inquiries can be directed to the corresponding author.

## Author Contributions

All authors listed have made a substantial, direct, and intellectual contribution to the work, and approved it for publication.

## Conflict of Interest

The authors declare that the research was conducted in the absence of any commercial or financial relationships that could be construed as a potential conflict of interest.

## Publisher’s Note

All claims expressed in this article are solely those of the authors and do not necessarily represent those of their affiliated organizations, or those of the publisher, the editors and the reviewers. Any product that may be evaluated in this article, or claim that may be made by its manufacturer, is not guaranteed or endorsed by the publisher.
